# Biomechanical gait analysis in sheep: kinematic parameters

**DOI:** 10.3389/fbioe.2024.1370101

**Published:** 2024-05-20

**Authors:** Bruna Silva, Filipa João, Sandra Amado, Rui D. Alvites, Ana C. Maurício, Bárbara Esteves, Ana C. Sousa, Bruna Lopes, Patrícia Sousa, Juliana R. Dias, António Veloso, Paula Pascoal-Faria, Nuno Alves

**Affiliations:** ^1^ Centre for Rapid and Sustainable Product Development (CDRSP), Polytechnic of Leiria, Marinha Grande, Portugal; ^2^ Associate Laboratory for Advanced Production and Intelligent Systems (ARISE), Porto, Portugal; ^3^ CIPER—Biomechanics and Functional Morphology Laboratory, Faculty of Human Kinetics (FMH), University of Lisbon, Lisbon, Portugal; ^4^ Centro de Estudos de Ciência Animal (CECA), Instituto de Ciências, Tecnologias e Agroambiente da Universi-dade do Porto (ICETA), Porto, Portugal; ^5^ Departamento de Clínicas Veterinárias, Instituto de Ciências Biomédicas de Abel Salazar (ICBAS), Universidade do Porto (UP), Porto, Portugal; ^6^ Associate Laboratory for Animal and Veterinary Science (AL4AnimalS), Lisboa, Portugal; ^7^ Cooperativa de Ensino Superior Politécnico e Universitário (CESPU), Porto, Portugal; ^8^ University of Trás-os-Montes and Alto Douro (UTAD), Vila Real, Portugal; ^9^ Department of Mathematics, School of Technology and Management, Polytechnic of Leiria, Leiria, Portugal; ^10^ Department of Mechanical Engineering, School of Technology and Management, Polytechnic of Leiria, Leiria, Portugal

**Keywords:** sheep model, gait analysis, 3D kinematic, spatio-temporal parameters, joint angular displacement, biomechanics

## Abstract

Animals have been used as models to help to better understand biological and anatomical systems, and pathologies in both humans and non-human species, and sheep are often used as an *in vivo* experimental model for orthopedic research. Gait analysis has been shown to be an important tool in biomechanics research with clinical applications. The purpose of this study was to perform a kinematic analysis using a tridimensional (3D) reconstruction of the sheep hindlimb. Seven healthy sheep were evaluated for natural overground walking, and motion capture of the right hindlimb was collected with an optoelectronic system while the animals walked in a track. The analysis addressed gait spatiotemporal variables, hip, knee and ankle angle and intralimb joint angle coordination measures during the entire walking cycle. This study is the first that describes the spatiotemporal parameters from the hip, knee and ankle joints in a tridimensional way: flexion/extension; abduction/adduction and inter/external rotation. The results of this assessment can be used as an outcome indicator to guide treatment and the efficacy of different therapies for orthopedic and neurological conditions involving the locomotor system of the sheep animal model.

## 1 Introduction

Gait is a fundamental function of any animal. It is required to forage for food sources, pursue prey, avoid stressful environments, and even for reproductive behavior. Therefore, gait analysis is an accurate, quantitative, and objective method to record limb function during everyday activities and how it is affected by disease (Simon, 2004; Kim and Breur, 2008).

Because sheep are similar in size to humans, and the age ratio is well established between the two species, results are easily translated and replicated in surgical procedures, sample collection, and imaging information. Other advantageous aspects about the use of sheep for research are the fact that they are docile, easily available, have low maintenance and feeding costs, as well as being accepted as research animals with fewer ethical restrictions. Some downsides are that sheep have herding behavior, they are commonly distrustful of humans, and therefore large spaces are needed for the manifestation of their gregarious behavior (Alvites et al., 2021).

Gait analysis is a non-invasive method. It can provide objective data without changing the conditions of the study being done. Analyzing the gait cycle has been shown to be an important tool in biomechanics research and its clinical application. The results of this assessment can be used as an outcome indicator to guide treatment and the efficacy of different therapies (Kim and Breur, 2008; Silva et al., 2019). Some studies have employed gait analysis as a method to assess the progression of diseases, healing process and for tissue engineering (Duda et al., 1998; Seebeck et al., 2005; Tapper et al., 2006; Hébert-Losier et al., 2008; Mora-Macías et al., 2015; Blázquez-Carmona et al., 2021; 2023). The fact that gait analysis has these applications increases the relevance of this work. Yet, improving comprehension of abnormal gait necessitates establishing parameters for what constitutes normal gait. The term “normal” should be understood within the context of variations linked to factors such as sex, age, and body geometry. Furthermore, in animals, morphological variations linked to breeds should also be taken into account (Faria et al., 2014).

Spatiotemporal parameters were described for the hind and forelimbs of sheep of different ages, concluding that sheep of two different ages walking to a constant velocity, had similar kinematic data between sides, and exhibit some differences in kinematic variables that may be age-related or body size (Faria et al., 2014). The limits of normal motion within the intact ovine knee joint were also outlined, thus furnishing baseline data for future studies, including the exploration of joint motion in ligament-transected joints of the same subjects (Tapper et al., 2006). The ranges of movement for flexion, extension, external rotation and translations of the sheep’s knee were calculated for walking, inclined walking and even trotting (Tapper et al., 2006). The sheep were subjected to surgery to implement stainless steel bone plates where markers were later placed for kinematic analysis. Thus, the kinetic and kinematic values were described, but through an invasive evaluation method (Tapper et al., 2006). Another study examined the relationships between kinematics, knee kinetics, contact pressures, and ligament loads for multiple ADLs and, as expected, the results demonstrated that declining gait decreases flexion of the knee at hoof strike and increases flexion during push off (Spatholt et al., 2023).

Recently, Pablo (Blázquez-Carmona et al., 2023) have conducted in-depth exploration of gait analysis as an effective means to mechanically monitor bone regeneration in critical-sized defects within tissue engineering applications. The authors demonstrated that gait analysis effectively tracks the regeneration of critical defects treated by tissue engineering. Nevertheless, they reported the greater surgical simplicity that the direct replacement of a fragment implies, gait recovery is slower than in other bone regeneration processes, such as bone transport. The authors also concluded that a force platform proves to be a valuable, cost-effective tool in the fields of traumatology and tissue engineering because it offers indirect, non-invasive, continuous, and quantitative *in vivo* monitoring of the regeneration process without necessitating complex biomechanical and kinematic expertise, thus making it readily applicable in clinical settings (Blázquez-Carmona et al., 2023).

Furthermore, lates research trends in gait analysis used wearable sensors and machine learning reporting that wearable sensors which provide a convenient, efficient, and inexpensive way to collect data and machine learning methods which enable high accuracy gait feature extraction for analysis (Muro-de-la-herran et al., 2014; Saboor et al., 2020; Slijepcevic et al., 2021; Harris et al., 2022; Sethi et al., 2022; Slemenšek et al., 2023).

Previous studies have already performed a kinematic analysis of the sheep’s gait, but only considering the sagittal plane (Safayi et al., 2015; Diogo et al., 2021). Diogo et al. (2021) even compared the results obtained through 2D and 3D analysis. The fact that the amount of movement is greater in the sagittal plane makes this the focus of movement analysis. However, failure to analyze kinematic data in the transverse and frontal planes limits the understanding of joint movement in 3D. The purpose of the present study is to perform a detailed segmental kinematic analysis during walking using a tridimensional reconstruction of the sheep hind limb, regarding the morphology and the movement of the thigh, shank, and foot. It is hoped that this data may provide a first approach to standard values with which abnormal walking joint kinematics may be compared. João et al. (2010) have already developed this methodology for rat using the rat animal model. The authors described the kinematics of the rats’ hind limbs during their gait, considering the three planes of motion. The data was collected with an optoelectronic system of 6 cameras Qualisys (0qus-300) operating at a frame rate of 200 Hz. The rats were previously shaved and 7 reflective markers with 2 mm diameter were attached to 7 bony prominences (João et al., 2010). In the present study, the methodology used will be similar.

### 1.1 Kinematic parameters

Depending on the locomotion speed and environmental conditions, quadrupeds can exhibit various gait patterns such as walking, trotting, pacing, bounding, *etc.* Moreover, at low speeds, different quadruped species exhibit distinct walking patterns (Owaki et al., 2013). These types of gaits can be divided into two different categories: symmetrical gaits (e.g., pace, walk or trot), which occur when the footfalls of both the fore pair and the hind pair are evenly spaced in time, and asymmetrical gaits (e.g., gallop and bound), which occur when the movements of at least one pair are unevenly spaced in time (Abourachid, 2003). Sheep, like horses, demonstrate a lateral-sequence walk, where their feet touch the ground in the sequence of right hind, right fore, left hind, and left fore (Owaki et al., 2013).

The gait cycle is the time between hoof strike to hoof strike, from the same limb (Rifkin et al., 2019; Silva et al., 2019). This cycle is divided in the stance and swing phases. The stance phase is the part of the gait cycle that begins as soon as the hoof contacts the floor and ceases when the hoof is lifted from the floor and starts its forward movement. The swing phase begins at the onset of forward movement and terminates as the hoof strikes the floor. The initial hoof floor contact (initial contact, IC) starts the stance phase and immediately when the hoof is lifted from the floor (toe off, TO), the swing phase starts (Rifkin et al., 2019; Diogo et al., 2021).

The step length was considered as the distance between the heel point of one foot to the heel point of contralateral one (Carr and Dycus, 2016). Stride Length is the distance measure parallel to the line of progression between the posterior heel points of two consecutive footfalls of a given extremity (Carr and Dycus, 2016; Rifkin et al., 2019; Silva et al., 2019; Diogo et al., 2021). Stride width is the lateral distance between the point of contact of the two pelvic limbs, perpendicular to the direction of the movement. Gait velocity is the gait distance divided by the gait time (Rifkin et al., 2019).

Abourachid, (2003) argues that the analysis of the gait of quadrupeds must go beyond the analysis of the stride. He considers: the F lag (Forelimb delay) that represents the time interval between movements of the front limbs; the H lag (Hindlimb delay) that refers to the time interval between movements of the hind limbs and the P lag (Pair delay): Indicates the time interval between movements of ipsilateral limbs, i.e., between limbs on the same side of the body. All are expressed as a percentage of the duration of the movement cycle.

These parameters allow for the characterization of different types of gaits based on the temporal delays between limb movements, providing a detailed and accurate representation of locomotion patterns in quadrupeds (Abourachid, 2003).

In symmetrical gaits, it is defined that the time lags between the two feet of the pairs (F lag and H lag) are equal, constituting 50% of the cycle duration. The coordinated movement of ipsilateral feet in the pace results from the time lag between the actions of the fore and hind pairs (P lag). In pace, where P lag equals 100% of the cycle duration, the two right feet and two left feet move simultaneously (Abourachid, 2003).

Another variable considered is the duty factor: a term used to describe the fraction of the movement cycle during which a limb remains in contact with the ground. It is the proportion of the total time of a movement cycle in which a limb is in contact with the ground. The duty factor is similar for all four limbs in symmetrical gaits, but may vary in asymmetrical gaits, indicating a shift in the distribution of body weight during different types of locomotion (Abourachid, 2003).

## 2 Methods

### 2.1 Experimental animals

The study involved 7 healthy adult Merino female sheep. Each one was examined by a veterinarian, who certified that they were all healthy and not pregnant. The animals were between 5 and 6 years old and weighed between 40 and 60 kg (with an average weight of 47.25 ± 4.27 kg). They were being kept at the Clinical and Veterinary Research Center of Vairão (CCIVV) and were fed hay and feed and had free access to water according to their metabolic needs.

The activities described in this work were carried out in an integrated way in the Bone2Move project: development of experimental techniques and modeling methodologies for the evaluation of 4D implants in bone defects in the ovine model (IC&DT Project 02/SAICT/2017). This study followed high ethical values, so the wellbeing of the animals was always ensured throughout the tests. To this end, all activities involving animal models were previously approved by the *Organismo para o Bem Estar Animal (ORBEA) from ICBAS-UP (Projeto 2880/2015)*.

### 2.2 Motion capture

On the data collection day, the animals were not fed in the morning, to stimulate locomotion and get more accurate kinematic results. The feed was used as incentive. The data was collected through 6 infrared cameras (Qualisys Miqus 3, Qualisys AB, Sweden), three on each side of a corridor where the gait is performed, to sense the markers placed on both hindlimbs of the sheep. The kinematic data was collected using (Qualisys Track Manager), (Qualisys AB, Sweden) operated at a frame rate of 100 Hz.

To obtain more accurate results, the seven sheep were sheared the day before data collection. That same day, the animals were taken on a trial/reconnaissance walk through the corridor used for the test, so that they became aware of the surrounding environment.

**FIGURE 1:**
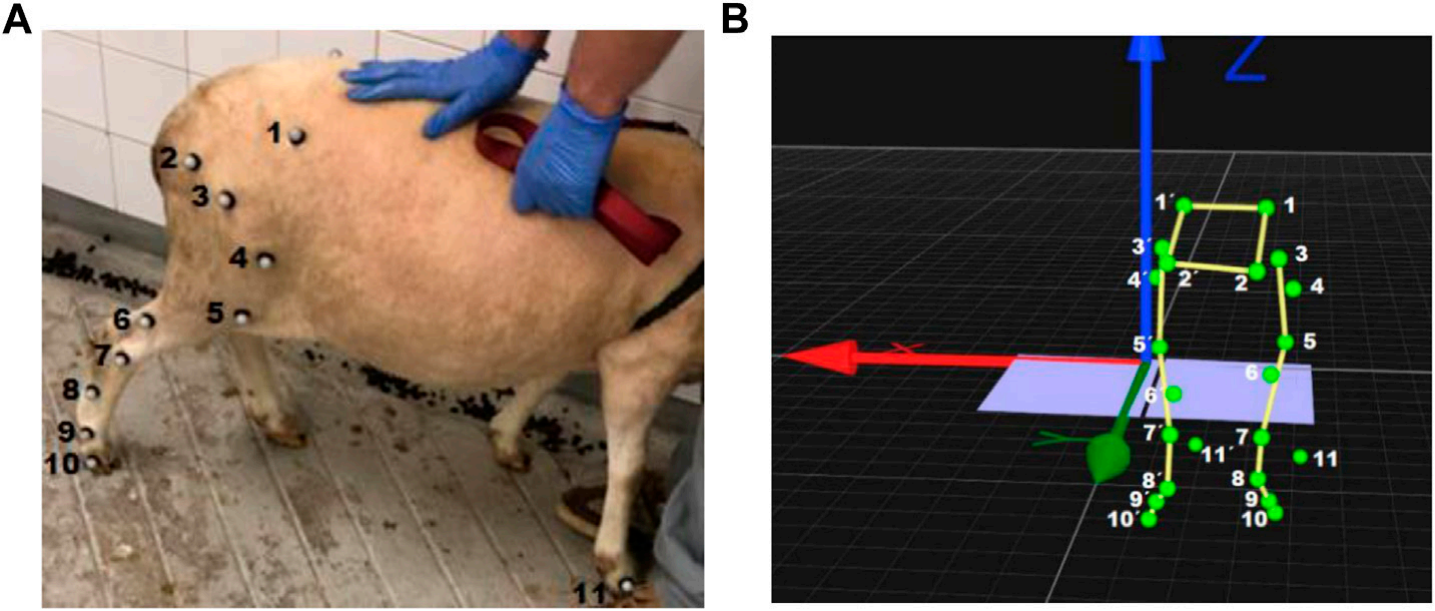
**(A)** Anatomical landmarks where the retroreflective markers were placed (right hind limb) and **(B)** Image taken from Qualisys Track Manager 2020.3, corresponding each green dot in the 3D model to the position of the retroreflective markers placed on both hind limbs: 1. Coxal tuberosity of the iliac wing; 2. Ischial tuberosity; 3. Greater trochanter of the femur; 4. Craniolateral aspect of the femoral shaft; 5. Femorotibiopatelar joint; 6. Caudoproximal aspect of the tibial shaft; 7. Base of the calcaneus; 8. Caudopoximal aspect of the IV metatarsal; 9. Metatarsophalangeal joint; 10. Lateral aspect of the distal phalanx and 11. Lateral aspect of the distal phalanx of the right forelimb.

22 spherical reflective markers were placed on both hindlimbs by the same examiner, using double face tape and superglue (Figure 1). Out of the 22 markers, 10 were place on each side of the pelvis with specific anatomical references: coxal tuberosity of the iliac wing; ischial tuberosity; greater trochanter of the femur; craniolateral aspect of the femoral shaft; femorotibiopatelar joint; caudoproximal aspect of the tibial shaft; base of the calcaneus; caudopoximal aspect of the IV metatarsal; metatarsophalangeal joint and lateral aspect of the distal phalanx. The remaining 2 markers were placed on the right and left forelimbs on the lateral aspect, serving an indicator of the ground contact with that limb.

After marker placement, the test began, with the guidance of the same person for all the tests and with the help of a leash, without limiting the animals’ freedom of movement.

### 2.3 3D model and kinematic analysis

A 3D model of the pelvis and both hindlimbs was built using Visual 3D software (Visual 3D, C-motion Inc., United States).

In view of the importance of an anatomically meaningful kinematics of sheep hindlimb, a more functional approach should be considered with the goal of allowing the description of the relative movement between two contiguous bony segments: one proximal and another distal. The absolute and segmental reference systems were previously described (João et al., 2010).

**FIGURE 2:**
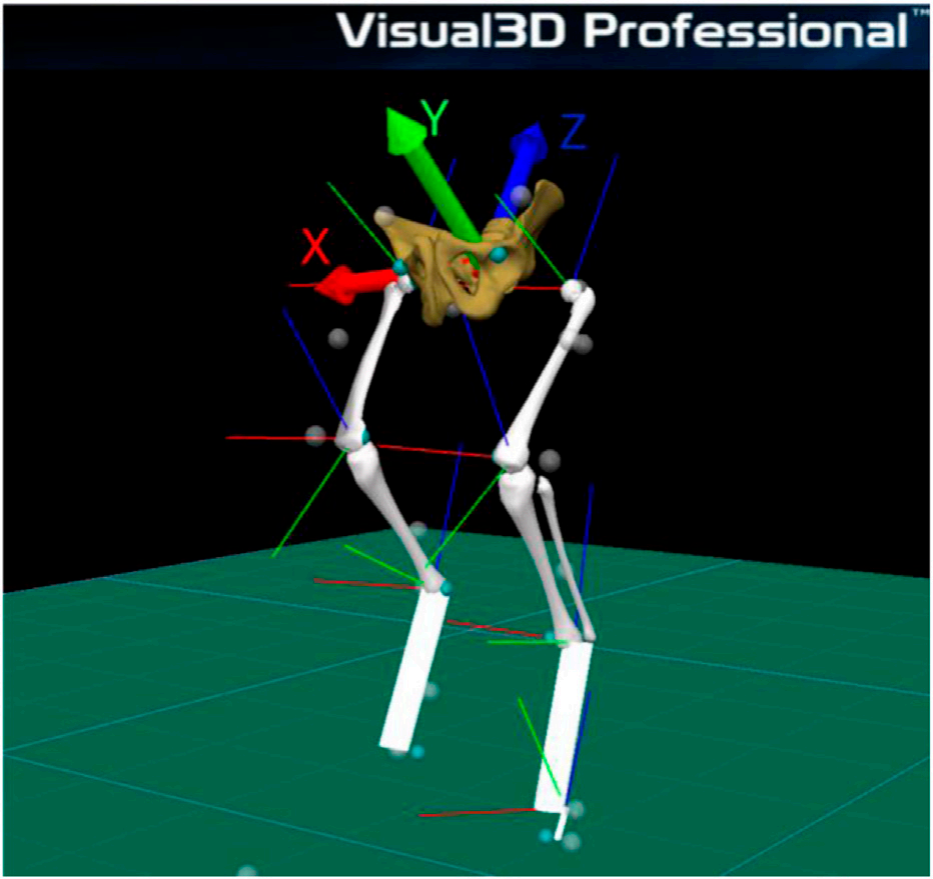
Tridimensional coordinate system.

In the lateral aspect of third phalanx, metatarsophalangeal joint, tibiotarsal joint and femorotibiopatellar joint, distance was respectively, 3.10, 2.70, 2.90 e 3.70 cm. Five body segments were reconstructed: pelvis, thigh, shank and foot metatarsus and hoof using Visual 3D software for biomechanics modeling. Each segment has an embedded tridimensional coordinate system. Each segmental reference system has its origin in the segment’s center of mass and is oriented in the following directions: X positive (medio-lateral axis of rotation - flexion/extension), Y positive (anterior-posterior axis of rotation - abduction/adduction), and Ζ positive (longitudinal axis of rotation - axial rotation), as shown in Figure 2.

The hip joint angle was defined as the angle oriented rostrally between the pelvis and femur. Pelvis orientation was defined by the line joining the markers at the coxal tuberosity of the iliac wing (Figure 1 (1)) and ischial tuberosity (Figure 1 (2)), while femur orientation was given by the segment between markers at the greater trochanter and lateral aspect of the knee. Knee angle was defined by the angle oriented caudally between the femur and the shank, with the latter construed by the markers at the knee and lateral malleolus (Figure 1 (5)). Last, the ankle joint was defined as the angle formed between the leg and hindfoot, with the latter segment defined by markers at the lateral malleolus and caudoproximal aspect of the IV metatarsal (Figure 1 (7)).

### 2.4 Statistical analysis

Each sheep walked for around 3 min for data collection purposes, without speed constraints. However, only cycles frames where the locomotion was firm in a straight line and in a comfortable way, were used for the final data analysis. Therefore, hesitations, such as dragging the limbs, or running cycles led to the rejection of the corresponding data. The average speed was calculated using the average values recorded for each sheep ant it was 1.16 ± 0.29 m/s. A total of 85 gait cycles of lateral-sequence walk were analyzed and the spatio-temporal parameters as well as the joint angular displacements were normalized to the total duration of the gait cycle. The joint angle-angle cyclograms for the three hindlimb joints were plotted to allow qualitative inspection of interjoint coordination. Average values were calculated by averaging the values for the 85 analyzed cycles. Despite the selection of sheep being based on their similar characteristics (breed, body mass, and age), it is important to consider the existence of inter-sheep differences.

## 3 Results

### 3.1 Spatio-temporal parameters

Spatio-temporal kinematic parameters of gait were calculated for the left and right hindlimbs (Table 1), and for left and right forelimbs (Table 2) The parameters are: cycle time, stance time, swing time, step length, step time, stride length, stride width, double limb support time and gait speed.

**TABLE 1:** Spacio-temporal kinematic parameters for hindlimb.

Variable	Mean	
Cycle Time (s)	Left	Right
	0.602 ± 0,114s	0.628 ± 0,100s
Stance Time (s)	Left	Right
	0.373 ± 0,047s (61%)	0.378 ± 0,091s (60%)
Swing Time (s)	Left	Right
	0.236 ± 0,114s (39%)	0.252 ± 0,037s (40%)
Step Length (m)	Left	Right
	0.398 ± 0.099 m	0.340 ± 0.120 m
Step Time (s)	Left	Right
	0.313 ± 0.068s	0.298 ± 0.064s
Stride Length (m)	0.695 ± 0.098 m	
Stride Width (m)	0.191 ± 0.036 m	
Double Limb Support Time (s)	0.146 ± 0.089s	
Speed (m/s)	1.135 m/s	

**TABLE 2:** Spacio-temporal kinematic parameters for forelimb.

Variable	Mean	
Cycle Time (s)	Left	Right
	0.563 ± 0,124s	0.593 ± 0,112s
Stance Time (s)	Left	Right
	0.359 ± 0,087s (62%)	0.354 ± 0,090s (59%)
Swing Time (s)	Left	Right
	0.231 ± 0,036s (38%)	0.247 ± 0,043s (41%)
Step Lengh (m)	Left	Right
	0.345 ± 0.063 m	0.323 ± 0.089 m
Step Time (s)	Left	Right
	0.296 ± 0.061s	0.289 ± 0.058s
Stride Lengh (m)	0.650 ± 0.147 m	
Stride Width (m)	0.162 ± 0.036 m	
Double Limb Support Time (s)	0.134 ± 0.076s	
Speed (m/s)	1.126 m/s	

#### 3.1.1 Hindlimbs

For the hindlimbs, the gait cycle described by the left limb took about 0.602 ± 0.114 s, with the stance phase being about 61% (0.373 ± 0.047 s) and the swing phase about 39% (0.236 ± 0.114 s). The gait cycle for the right limb took about 0.628 ± 0.100 s. The stance phase corresponds to about 60% of the cycle (0.378 ± 0.091 s) and the swing phase corresponds to about 40% (0.252 ± 0.037 s).

The distance between the successive contacts of the left hoof was 0.398 ± 0.099 m being performed in 0.313 ± 0.068 s. Two successive contacts of the right hoof marked 0.340 ± 0.120 m and took 0.298 ± 0.064 s. The length of the stride was 0.695 ± 0.098 m and the width of the stride was 0.191 ± 0.036 m. The double limb contact time was 0.146 ± 0.089 s, while during 0.019 ± 0.012 s there was no contact of either limb with the ground. The average speed of the sheep’s hind limbs was 1,135 m/s.

#### 3.1.2 Forelimbs

For the forelimbs, the gait cycle described by the left limb took about 0.563 ± 0.124 s, the stance phase being about 62% (0.359 ± 0.087 s) and the swing phase about 38% (0.231 ± 0.036 s). The gait cycle for the right forelimbs took about 0.593 ± 0.112 s. The stance phase corresponds to about 59% of the cycle (0.354 ± 0.090 s) and the swing phase corresponds to about 41% (0.247 ± 0.043 s).

The distance between the successive contacts of the left hoof was 0.345 ± 0.063 m being realized in 0.296 ± 0.061 s. Two successive contacts of the right hoof marked 0.323 ± 0.089 m and took 0.289 ± 0.058 s. The stride length of the forelimbs was 0.650 ± 0.147 m and the stride width was 0.162 ± 0.036 m. The double limb contact time was 0.134 ± 0.076 s, and the average speed of the sheep’s forelimbs was 1,126 m/s.

### 3.2 Joint angular displacement

The joint angular displacement of the sheep model was calculated from a standing position (Figure 2).

**FIGURE 3:**
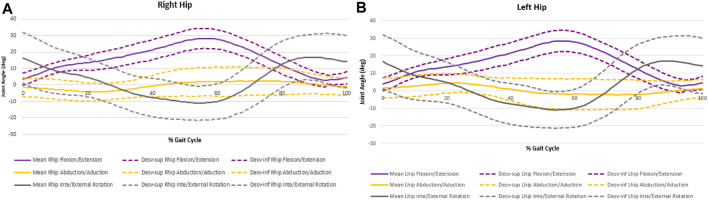
Hip angular displacement for right hind limb **(A)** and left hind limb **(B)**.

Only hindlimbs were considered for joint angular displacement analysis, and the curves presented by the right and left limbs are quite similar (Figure 3), with some variations justified by the methodology of motion capture and subsequent analysis. For this reason, only data from one limb was considered for the analysis of joint angular displacement over the gait cycle.

**FIGURE 4:**
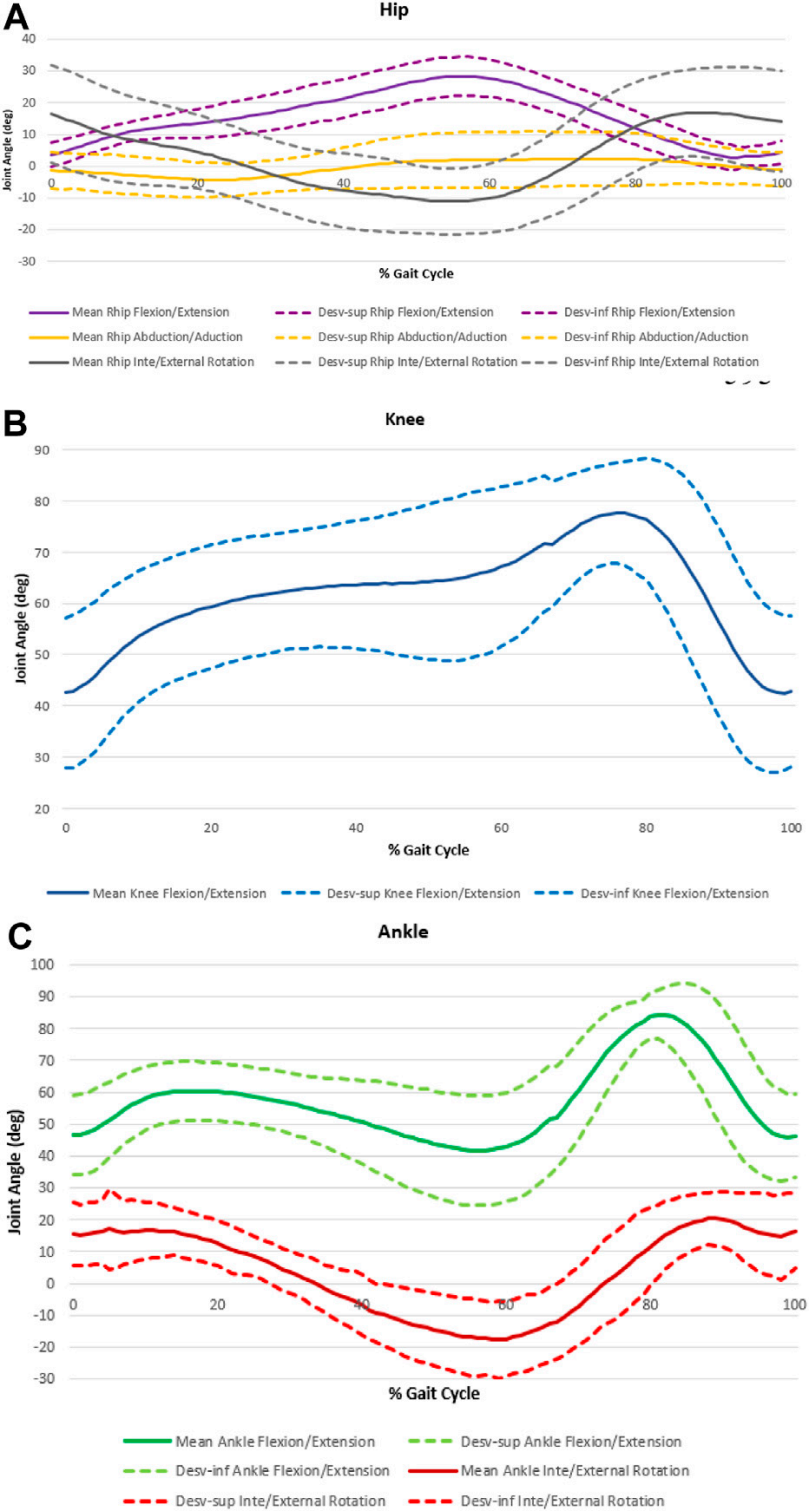
Joints angle amplitudes over the cycle for hip **(A)**, knee **(B)** and ankle **(C)**.

The variation of the angle amplitudes of the various joints, according to the three axes, over the cycle, is shown in Figure 4.

The Figure 4 and Table 3 show the angle variation curves and the values of the main events—IC, TO, minimum and maximum value in the stance phase (Stancemin and Stancemax) and minimum and maximum value in the swing phase (Swingmin and Swingmax).

**TABLE 3:** Joint Angles variation of the main events.

	Ankle		Knee		Hip	
	Flexion/Extension	Inversion/Eversion	Flexion/Extension	Flexion/Extension	Abduction/Aduction	Internal rotation External rotation
IC	45,448˚ ± 11,786°	13,682˚ ± 10,400°	46,869˚ ± 14,904°	5,485˚ ± 7,383°	−4,439˚ ± 4,521°	−7,765˚ ± 7,109°
STANCEmin	38,030˚ ± 12,081°	−20,501˚ ± 9,439°	46,869˚ ± 14,904°	5,485˚ ± 7,383°	−4,464˚ ± 4,624°	−8,423˚ ± 7,785°
STANCEmax	56,225˚ ± 10,024°	15,184˚ ± 9,605°	68,646˚ ± 14,615°	31,899˚ ± 7,737°	1,658˚ ± 8,468°	2,129˚ ± 6,864°
TO	40,804˚ ± 13,951°	−19,954˚ ± 9,698°	69,387˚ ± 14,746°	31,231˚ ± 8,224°	1,336˚ ± 7,892°	2,117˚ ± 6,921°
SWINGmin	40,804˚ ± 13,951°	−19,954˚ ± 9,698°	46,384˚ ± 15,937°	4,135˚ ± 7,384°	−5,438˚ ± 5,255°	−14,950˚ ± 6,535°
SWINGmax	80,668˚ ± 11,541°	15,207˚ ± 16,618°	80,622˚ ± 11,784°	31,231˚ ± 8,224°	1,336˚ ± 7,892°	2,117˚ ± 6,921°

The curves representing the angular variation over the cycle in the knee and ankle joints are quite similar. At IC, the knee is already in flexion (46,869° ± 14,904°), increasing the flexion movement throughout the stance phase. The ankle begins the dorsiflexion cycle (45,448° ± 11,786°), passing the plantar flexion during the stance phase. At the time of TO, the ankle dorsiflexion movement begins again (13,682° ± 10,400°), with the mid-swing phase reaching the knee flexion peak and the ankle dorsiflexion peak (69,387° ± 14,746° and 80,668° ± 11,541°, respectively).

Considering the rotational movements, the pattern is like both the level of the hip and the ankle joint. Although the angular variations throughout the cycle are reduced, both joints in the IC show slight internal rotation (IR) (13,682° ± 10,400 of inversion for the ankle and −7,765° ± 7,109° of IR for the hip) which during the support phase becomes external rotation (ER) (−19,954° ± 9,698° of eversion for the ankle and 2,117° ± 6,921° of ER for the hip). At the time of TO, the ER is maintained, and after 80% of the cycle, a new IR is performed, both hip and ankle joint.

The abduction/adduction movement at the hip has the smallest amplitude variation. The joint starts with an abduction movement (−4,439° ± 4,521° at the IC) until the end of the stance phase. In the mid-swing phase start the adduction movement until the end of the gait cycle.

In the IC, the ankle presents 45,448° ± 11,786° of dorsiflexion and 13,682° ± 10,400° of inversion and at the moment of TO. The knee starts the cycle with 46,869° ± 14,904° of flexion and the TO angle is 69,387° ± 14,746° of flexion.

### 3.3 Cyclograms

**FIGURE 5:**
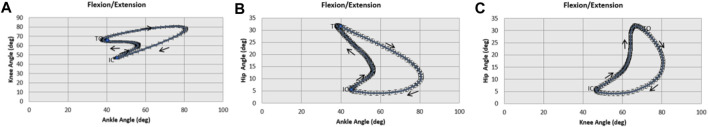
Intralimb joint angle coordination. **(A)** Illustrates the coordination between knee and ankle during gait cycle. **(B)** Illustrates the coordination between hip and ankle during gait cycle. **(C)** Illustrates the coordination between hip and knee joint during gait cycle. IC: initial contact, TO: toe-off, black row: gait cycle progression direction.

Figure 5 illustrates the intralimb joint angle coordination. These cyclograms display simultaneous motion of two joints allowing the comparison of angular displacement between them during the gait cycle. Between the IC and the TO, following the direction of thin rows, is the stance phase. Pursuing the same direction, the swing phase is between the TO and the IC.

The knee-ankle cyclogram displays that right before the TO the ankle reaches its maximum extension whilst peak knee extension occurs at IC. The ankle flexes between the IC and midstance. At this moment, the ankle begins its extension until right before the TO. On the other hand, knee flexion increases throughout the entire stance phase. Both joints start to flex after the TO, reaching their maximum flexion at midswing. From this moment, they start their extension until the next IC.

The hip-ankle cyclogram illustrates that in both joints the maximum extension occurs right before the TO. During the stance phase the ankle joint goes through a period of flexion first and then through a period of extension. While there is an extension of the hip during all the stance phases. From TO both joint flex until midswing. At this point, the hip continues to increase its flexion angle reaching its maximum flexion right before the IC. While ankle flexion decreases through the remaining swing phase.

The hip-knee cyclogram shows that at IC the knee reaches its maximum extension whilst the hip is fully extended right before the TO. From here on, the hip starts to flex and hits its maximum flexion right before the next IC. Whereas knee flexion has already been started after IC and continues to increase its flexion angle until midswing, where it peaks.

## 4 Discussion

Animals have been used as models to help us better understand biological and anatomical systems, and pathologies in both humans and non-human species (Kim and Breur, 2008). This type of research allows for new knowledge of diseases, which eventually can be applied in the corresponding treatments. These therapeutic approaches can be surgical or non-surgical. In surgical methods, there could be improvements in existing techniques or just new techniques. It also allows for a better forecast of results and prognosis (Kim and Breur, 2008).

Sheep are often used as an *in vivo* experimental model for orthopedic research (Kim and Breur, 2008). Some of the conditions that have been studied and treatment applied through this method are musculoskeletal pathologies, such as bone fractures, transport, lengthening and rupture of articular ligaments and limb lengthening (Duda et al., 1998; Seebeck et al., 2005; Tapper et al., 2006; Hébert-Losier et al., 2008; Mora-Macías et al., 2015; Blázquez-Carmona et al., 2021). They also have been applied in a variety of degenerative diseases, like osteoarthrosis and osteoporosis, muscular disorders, osteomyelitis, and neurological diseases. Most often these animal models are used to test the therapeutic efficacy of biomaterials/tissue engineering. (Kim and Breur, 2008; Blázquez-Carmona et al., 2023). Biomechanical gait analyses are a non-invasive method to study biomechanical parameters, and since gait assumes the most important task in the sheep routine, evaluation of such parameters should be considered for research and clinical applications.

Sheep with and without moderate spinal cord injury displayed distinct kinematics, suggesting the potential utility of evaluate novel neuromodulation devices. Specifically, pelvic limb hoof elevation during the swing phase emerged as a promising metric for assessing the impact of spinal cord injury and as a potential target for evaluating the efficacy of a therapy in correcting neurological deficits post-injury in future studies (Safayi et al., 2015). 3D stifle kinematics were employed to measure the impact of complete lateral meniscectomy in Suffolk-cross sheep. Kinematic irregularities were associated with early osteoarthritis severity in surgical models involving anterior cruciate ligament/medial collateral ligament transection in Suffolk-cross sheep (Faria et al., 2014).

There are already some studies that analyze the kinematics of rat gait (João et al., 2010; Amado et al., 2011), but little is still known about the gait kinematics of sheep that consider the 3 axes of movement. The kinematic results presented should provide direction for future gait analysis studies that evaluate the forelimb joint behavior, for a complete description of the sheep animal model. In the future, this experimental model may serve as an effective tool to evaluate and compare pathological gait patterns and improve our knowledge on kinematic features associated with different orthopedic and neurological conditions (Diogo et al., 2021).

In general, the stance and swing phases account for approximately 60% and 40%, respectively, of the gait cycle in healthy humans (Alvites et al., 2021). A systematic review about sheep gait biomechanics parameters reported a stance phase for hind limbs between 61.88%–68.89% and swing phase from 31.11%–38.85% and for the forelimb, the stance phase ranges between 58.86%–66.31% and swing phase from 41.49%–33.69%. In the systematic review (Silva et al., 2019), the distribution was 61% stance phase and 39% swing phase for the left hindlimb and 60% stance phase and 40% swing phase for the right hindlimb. For left forelimb the stance phase was 61% and the swing phase was 39% and 59% for right forelimb’s stance phase and 41% for swing phase. As can be seen, the values for the stance and swing phase are among the values indicated by the systematic review about sheep biomechanics and the values are like the values of human gait. Another study of sheep reported a mean of 68.89% for the hind limbs and 66.31% for the forelimbs in stance phase and 31.11% for the hind limbs and 33.69% for the forelimbs and in swing phase (Kim and Breur, 2008).

For the forelimbs, the gait cycle described by the left limb took about 0.563 ± 0.124 s and for the right limb took about 0.593 ± 0.112 s. The left stance phase being about 0.359 ± 0.087 s and the right stance phase being 0.354 ± 0.090 s while the left swing phase being about 0.231 ± 0.036 s and the right 0.247 ± 0.043 s. For the hindlimbs, the gait cycle described by the left limb took about 0.602 ± 0.114 s and the right took about 0.628 ± 0.100 s. The left stance phase being about 0.373 ± 0.047 s and the right stance phase 0.378 ± 0.091s while the and the left swing phase about 0.236 ± 0.114s and the right 0.252 ± 0.037 s (Diogo et al., 2021). results shows a gait cycle duration of 0.744 ± 0.450 s with a stance duration of 0.442 ± 0.310 s, a swing duration of 0.302 ± 0.300 s. Comparing the results, it appears that the results of the present study demonstrated smaller gait cycles, and consequently, smaller support and swing phases.

The stride length of the forelimbs was 0.650 ± 0.147 m and 0.695 ± 0.098 m for hindlimbs. When compared with the results of study (Diogo et al., 2021), with a stride length of 0.833 ± 0.560 m, it appears that the stride length was also shorter for both the forelimb and the hindlimb.

Besides reporting angular results of some selected instants of the gait cycle, presenting a continuous angle-time plot is important because the angular pattern in both stance and swing phases is also critical for gait assessment. More than angles in specific and limited instants in time, the complete pattern recognition and description provide important information concerning functional recovery assessment. Cyclograms provide relevant information about intralimb and interjoin coordination patterns (Goswami, 1998) and the study of these relationships, and their better understanding, represents more important kinematic data for the functional recovery of the sheep’s gait.

When we analyze joint kinematics data, we should take into consideration that the results are also related with neural and muscle function, thus being relevant to perform a comprehensive description of the movement of individual muscle action within the joint or the joints it crosses. The dynamics of muscle functions are usually examined by indirect assessment of muscle length change based on kinematics (Winter 2009) and moment muscle arms or estimates of fascicle length change that must consider in-series elastic stretch of a tendon and a simplification of the effects of muscle architecture. 3D movement patterns, even though more complex, are also more realistic, and are crucial not only for understanding the neuromuscular control of said movement patterns, but also for the principles of musculoskeletal design, which are vital for movement production (Biewener, 2002). For an accurate and realistic understanding of a movement pattern and its changes, combining kinematic data kinetics and electromyography would be ideal. Considering that animal models have a high potential of achieving and representing the various gait variability aspects and their non-linear interactions, the results of this study can very well represent a solid starting point for a muscle modeling approach on future cases.

Also, kinetic parameters should be considered, taking into account the studies already conducted by (Duda et al., 1998; Seebeck et al., 2005; Spatholt et al., 2023). During the sheep’s gait cycle, the interaction between vertical ground reaction forces (VGRF) and joint kinematics is crucial for the animal’s efficient movement. At the moment of IC, the VGRF is low, but as the sheep’s body weight is transferred to the paw in contact with the ground, the VGRF gradually increases (Duda et al., 1998; Spatholt et al., 2023). Simultaneously, the hip and knee joints extend, providing stability and support to the limb and the sheep’s body weight. However, the ankle flexes between IC and the middle of the support phase. In the middle of the support phase, the sheep carries all its weight on the paws that are on the ground, and the VGRF reaches its peak. At this point, the VGRF of the hind limbs is about 60% of the sheep’s body weight (Duda et al., 1998; Spatholt et al., 2023). As the TO moment approaches, the sheep begins to lift its legs off the ground with a simultaneous flexion in the hip, knee, and ankle extension. This combination allows for TO while the body weight is transferred to the other paws. During the swing phase, the joints remain flexed (with the knee reaching its maximum flexion during midswing and the hip reaching its maximum flexion just before the next IC), allowing the paws to move forward to start the next gait cycle. Meanwhile, the weight of the sheep’s body is supported by the other paws in contact with the ground (Duda et al., 1998; Spatholt et al., 2023).

A study created standardized 3 mm diaphyseal bone defects in the right tibia of 64 female sheep and stabilized them with either a rigid monoliteral external fixator or a more flexible variant. Over a 9-week healing period, gait parameters were measured using a pressure-sensitive platform, and interfragmentary movements at the fracture site were monitored. The results showed that ground reaction forces were strongly related to the course of callus mineralization and thus directly reflected the recovery of stiffness at the fracture site. Reduced levels of loading frequencies that may affect bone healing persist to 9 weeks postoperatively. These findings suggest that ground reaction forces and their changes over time can be useful indicators for monitoring fracture healing progress and may provide insights into the effectiveness of the different fixation methods utilized (Seebeck et al., 2005).

As forces and moments generated in the bones during walking have direct and indirect effects on the kinematics of adjacent joints, including the hip, knee, and ankle. This complex relationship highlights the importance of considering both internal forces and joint kinematics when studying animal movement. Similar to ground reactions, internal loads peak during the stance phase of walking for both the metatarsus and the tibia. Generally, bones are primarily loaded axially, with an increasing cranial shear force at their ends. Flexion moments in the metatarsus peak towards the ankle joint contact area, while in the tibia, they peak within the proximal portion of the bone due to massive muscles pulling distally and proximally (Duda et al., 1998). During the stance phase of walking, flexion moments in the tibia and metatarsus are influenced by various factors, including the magnitude of muscular forces and distribution of body weight. These moments directly impact the load and stability in the knee and ankle joints, influencing the kinematics of these joints as well as the hip. The magnitude of axial force shows differences of less than 10% throughout the gait cycle, while cranial and lateral force magnitudes differ slightly, around 20% (Duda et al., 1998).

Hind limb VGRF were evaluated during gait cycles. The average peak VGRF in hind limbs ranged from 34.5% to 50.0% of body weight. Ratios of peak VGRF in left to right hind limbs varied from 87.3% to 119.0%. Additionally, posterior, anterior, and medial ground reaction forces during different phases of walking were measured, showing variability in force distribution between limbs (Tapper et al., 2006). The study of kinetics in sheep gait should be considered in future studies for a more comprehensive analysis of the animals.

A possible source of error from kinematic signal processing relates to the use of skin markers that could introduce an error into the estimation of a segment’s length. Due to sheep quadrupedal nature, skin motion artifacts at the knee joint is the most relevant source of error when estimating hindlimb joint kinematics, due to a more extensive skin attachment from the proximal hindlimb to the lateral torso when compared to humans (Goswami, 1998)**.**

Using a treadmill can bring many advantages, including walking in a restricted area, accurate control of speed and gradient and the ability to capture repeated gait cycles. Furthermore, it’s possible to control the walking speed improving intra-session and inter-session reliability of measurements. Sheep’s walking locomotion has been previously reported with a velocity between 1.1 and 1.3 m/s (Diogo et al., 2021).

## 5 Conclusion

Since gait assumes the most important task in the sheep’s routine, the evaluation of such parameters should be considered. This study was the first that described the spatiotemporal parameters from the hip, knee and ankle joints in a tridimensional way: flexion/extension; abduction/adduction and inter/external rotation. The kinematic results should guide future gait analysis studies that evaluate the forelimb joint behavior, for a complete description of the sheep animal model. In the future, this experimental model may serve as an effective tool to evaluate and compare pathological gait patterns and improve knowledge on kinematic features associated with different orthopedic and neurological conditions.

## Data Availability

The original contributions presented in the study are included in the article/Supplementary material, further inquiries can be directed to the corresponding authors.
